# Women-centered cervical cancer prevention in a low-resource setting: a case study of The Lily Project, Nicaragua

**DOI:** 10.3389/fgwh.2026.1790330

**Published:** 2026-09-08

**Authors:** Kammi K. Schmeer, Sinem Esengen, Anielka Medina, Susan Cotton

**Affiliations:** 1 Department of Sociology, The Ohio State University, Columbus, OH, United States; 2 The Lily Project, Chicago, IL, United States

**Keywords:** gynecological health, Latin America, mobile health, sexual health, women's empowerment

## Abstract

Despite being preventable, cervical cancer is the fourth most common cancer among women worldwide. Barriers such as lack of quality care, social stigma, loss to follow-up, and limited knowledge of sexual health hinder access to cervical cancer screening and treatment, particularly among rural and low-income women. In this case study, we describe the Lily Project, a Nicaraguan-based cervical cancer prevention non-governmental organization working in a politically, socially and economically restrictive context. With no national HPV testing or vaccination program, and low quality, underfunded public health services, The Lily Project aimed to bolster cervical cancer screening and education for marginalized women in rural areas. Through multiple stakeholder collaboration, the organization implemented a women-centered approach that included mobile screening clinics and education to provide women with informed, private and female-conducted exams, results and follow up care. The Lily Project serves as a model of how women-centered care could be implemented in low-resource settings with particular attention to improving the quality of care for cervical exams for cervical cancer screenings among geographically and socially marginalized populations.

## Introduction

Cervical cancer is the fourth most common cancer among women worldwide, with the highest incidence and mortality rates occurring in low- and middle-income countries (LMICs). Inequalities in mortality are stark, with 94% of cervical cancer deaths occurring in LMICs (WHO, 2024). The leading cause of cervical cancer is chronic infection with human Papillomavirus (HPV), a common and widespread virus transmitted through sexual activity. As reported by the World Health Organization (2024), when left untreated, 95% of persistent HPV infections lead to cervical cancer (1).

The most effective way to prevent HPV infection is receipt of the HPV vaccine between the ages of 9 and 14 years (1), but globally, only 27% of girls have received the first dose of the vaccine (2). Further, while HPV vaccines are effective in dramatically reducing cervical cancer rates, they may not fully eliminate cervical cancer risk at the population level (3).

Thus, regular cervical cancer screenings using Papanicolaou (Pap) exams, visual inspection with acetic acid (VIA), and/or clinic or self-swab HPV tests for women starting at age 30 remain critical to preventing cervical cancer. Where available, cervical exams with HPV testing are more sensitive but also more costly and less specific than Pap tests (4). Both Pap and HPV test results require follow-up with women, including delivery of results after laboratory analysis, re-examination due to inconclusive findings, and further testing and treatment for positive cases. In some cases, using VIA followed by cryotherapy can be an effective treatment for reducing loss to follow-up by removing small, precancerous lesions within the same screening visit.

In LMICs, despite increased attention to cervical cancer screening, challenges remain in implementing effective screening programs. These include lack of cervical cancer and HPV awareness among women, their partners, and the larger community; the cost and time required to reach screening locations, particularly for marginalized populations; feelings of embarrassment and fear; and lack of family and spousal support for screening and treatment (5). In addition, low-income women often depend on services provided by under-resourced public health clinics that lack female providers, adequate sanitation, privacy before and during procedures, and reliable follow-up with results (5).

In this paper, we describe the Nicaraguan non-governmental organization (NGO) The Lily Project as one model for addressing several of these barriers to cervical cancer screening and treatment in low-income settings. While much attention has been paid to increasing uptake of services (and vaccines) and quality of testing for cervical cancer prevention (6), little research and interventions have focused on the conditions of the cervical exams (required by both Pap and VIA) that women “endure” throughout their lives. In many contexts, providers and programs operate within political, social, and economic settings that not only reduce rates of Pap and VIA screenings (either as first-line screening or after HPA-positive results) but also create difficult emotional and physical experiences for women during these screenings.

The Lily Project aimed to address both issues – improved access to and experiences during cervical cancer screenings—in the context of rural Nicaragua. Below, we describe the context within which The Lily Project operated, followed by descriptive results about their goals, processes, and results. We end by highlighting both potential successes and challenges faced by The Lily Project in implementing a “women-centered” model that improves cervical cancer screening experiences for marginalized women.

### Study context - Nicaragua

In Nicaragua, cervical cancer is the leading cause of cancer deaths among women aged 15 to 44 years and accounts for over 14,000 years lost to disability or death (7). The incidence rate of cervical cancer in Nicaragua is 21.2 per 100,000 women (8), markedly higher than rates in most Central, South, and North American countries.

While a national cervical cancer program was established by law in 1993, the Nicaraguan Ministry of Health has maintained an underfunded infrastructure for the primary method of cervical cancer screening—Pap testing (9). The program initially required annual Pap tests for women 15 years and older (10). By 2010, the national guidelines indicated the use of either Pap (women ages 25–64) or visual inspection (women ages 30–50) to be repeated every 3 years (11). HPV testing, considered the gold standard of screening, was assessed to be a cost-effective screening strategy for Nicaragua based on pilot studies (12). Further, self-collection kits were found to be socially acceptable and preferred by most women in a large pilot program (9). However, the government did not build on these pilots, and the Pap and VIA remained the required screenings designated by the Ministry of Health.

Within the larger context of LMIC research, Nicaragua has been cited as one of the places with a particularly low rate of follow-up Pap exams for HPV positive women (13). Several issues present barriers to the uptake of cervical cancer screenings in Nicaragua, including: the lack of education and misconceptions about Pap smears, HPV and cervical cancer; time and transportation barriers to attending clinic-based screenings; preferences for female providers who are often not available in public health clinics; and, lack of consistent follow-up by public health services in the delivery of results and treatment (14). These screening-specific issues sit within a broader societal context of gender-based structural violence and poverty (15–17), which further reduces women's access to adequate health care in Nicaragua.

It is within this context that we studied the processes and outcomes of the Lily Project, a women-centered cervical cancer prevention NGO based in Nicaragua, from 2015 to 2020. As a brief overview, The Lily Project (TLP) was established and led by a young Nicaraguan woman passionate about reducing cervical cancer following her mother's early death from the disease. The NGO was established to provide cervical cancer screening for marginalized women, specifically rural Nicaraguan women, through education and mobile clinics, with an all-female staff who lived in the targeted regions. The design and implementation of TLP were aided by two US professionals trained in business with a focus on social entrepreneurship, and by a gynecologist based in Managua. TLP operated on a relatively small budget with funding obtained through private donations.

The purpose of this paper is to present the results of an opportunistic study of TLP. This study was an organic effort that emerged from volunteer work by Ohio State University medical and graduate students and faculty. The Ohio State collaboration with TLP consisted of assisting with the development of an electronic data intake process, organizing a research database, and data analysis. Throughout this collaboration, we also documented the goals, processes and experiences of TLP. In this paper, we aim to share the model of women-centered care that we observed and obtained through informal interviews and data analysis with TLP staff and leadership. We highlight their attention to meeting multiple aspects of women's needs in designing their Pap and VIA screening process. This included educating women before and during the screenings, providing a private, clean, and supportive environment for the exams, and ensuring informed, reliable delivery of results and follow-up. All this was provided for free in local communities to guarantee access for rural women, and by an all-female staff who were familiar with the areas and had been trained in listening, emotional support, and leadership (as well as technical) skills.

While this is largely a descriptive rather than evaluative study, we hope that future programs may incorporate these elements, in particular, more attention to women's care needs before, during, and after the screenings and other cervical cancer prevention services. In the future, more formal evaluations of this women-centered approach, with attention to women's empowerment and broader social and health care issues, should be conducted.

## Methods

The methods for this case study included extensive fieldwork conducted by the first author with authors 3 and 4. This fieldwork included informal interviews with TLP leadership and staff, field observations, and analysis of internal TLP data and documents. The documents, including TLP website, educational materials, and intake forms were reviewed informally to gain a broad understanding of the goals of TLP. Informal interviews and discussions with TLP leaders, and on-the-ground observations, provided more detailed information about TLP process. The intake forms also contributed to the quantitative data analyzed here using Excel and R. Initially, data from paper intake forms was manually entered into an Excel spreadsheet as part of TLP protocol. As part of our collaboration, we helped move to an electronic data collection process (with the same form) that allowed for data to be automatically uploaded and saved to TLP database. Data accessed by our research team was de-identified and any new clients were added through an informed consent process. The institutional review board ethical approval for this project was received from Ohio State University and permission was provided by the Nicaraguan Ministry of Health.

## Results

### The Lily Project—goals and process

The vision of TLP was to provide women with privacy, dignity, and empowerment (through education) in the process of conducting cervical cancer screenings. This is consistent with feminist health care perspectives and what has been referred to as a “women-centered approach” in the fields of maternal health care (18, 19). We return to this idea of a women-centered approach in the discussion when reflecting on TLP goals and processes described below.

TLP emphasized four main quality promises to clients: (1) confidentiality of results and client information; (2) use of new, disposable materials for all exams; (3) guaranteed return of results within two weeks, if not immediately; and (4) that all data intake and gynecological exams would be performed by women (internal TLP documents). These promises were developed during TLP's strategic planning process based on observations of public health screenings in Nicaragua, informal interviews with rural women, and the lived experiences of TLP's Nicaraguan Executive Director and staff.

### Organization and training

The Lily Project delivery model was informed by best practices in cervical cancer screening and treatment established by the World Health Organization (WHO) and Nicaraguan Ministry of Health guidelines at the time. Because of the need for government approval and collaboration, TLP provided Papanicolaou (Pap) cytology and visual inspection with acetic acid (VIA), based on the age and other criteria established by the Ministry of Health. The goal was to provide these services for free, meeting the quality promises, and in rural communities.

TLP's human resources approach was to recruit and train local college-educated women (usually science or nursing majors) to conduct the screenings. This approach reduced the need for physicians at screening events and ensured that clients received exams conducted by women. Most staff had lived in the regions where they worked. Staff training was led by a gynecologist based in Managua and included both initial and refresher training to ensure the consistent provision of technically high-quality screenings.

Each TLP team consisted of one female team leader, two female support staff members, and one male driver. Team leaders conducted the exams with support from two trained female staff members. Capacity building for TLP leadership and staff also included training on how to provide respectful, supportive care to individual clients from the intake process through the delivery of results. Pap specimens were sent to a private laboratory for analysis.

Recognizing the need to work within existing political structures, TLP established agreements with the national and regional branches of the Ministry of Health to ensure government support. As part of this agreement, TLP used the Ministry of Health-developed intake forms and delivered a copy of the completed forms to the regional offices. This not only garnered government support but also ensured that TLP clients would have medical records in the Ministry of Health for future and collaborative follow-up. When possible, local public health centers provided space for TLP screening activities.

### Community collaboration

TLP identified and visited small rural communities and began working with community leaders well in advance to plan screening events. This process involved obtaining local-level government approval and building support through meetings with key community stakeholders and community-based educational events. Women were encouraged to attend upcoming screenings through public education sessions and flyers placed throughout the community.

**Figure 1:**
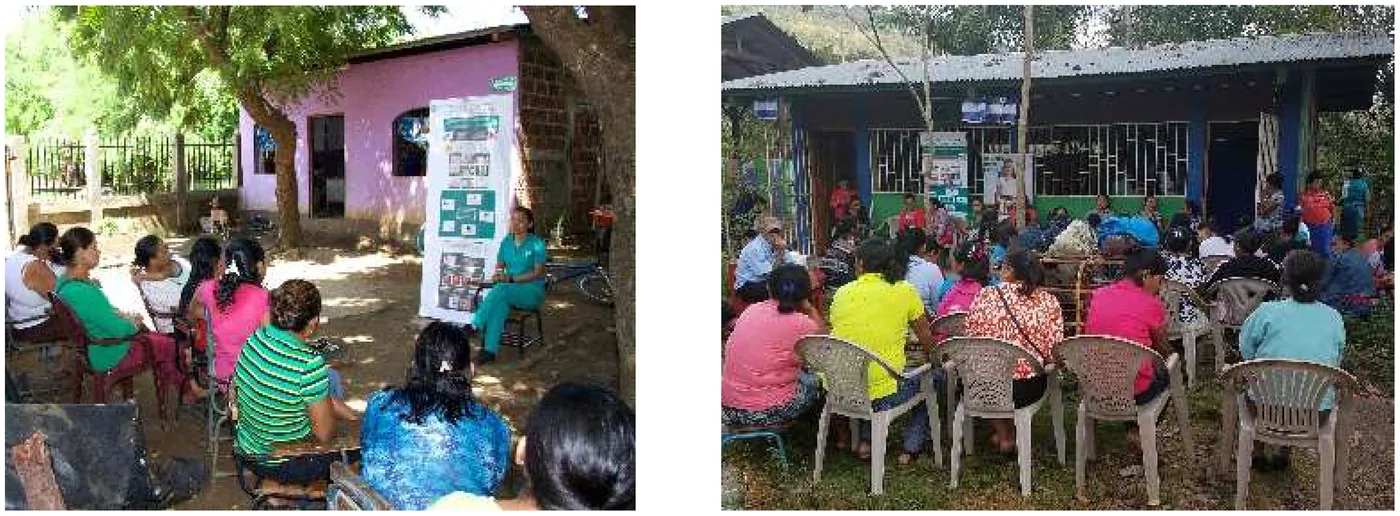
TLP community engagement. Photo credit: TLP 2019.

TLP's community-based education sessions primarily used posters with images, which staff explained verbally to groups of women with limited formal education. The sessions were open to the public, and women were encouraged to bring friends and family members, in part to help de-stigmatize discussions of cervical cancer prevention. TLP also developed additional materials and activities (such as bracelet-making) to help women and younger girls understand their bodies and reproductive processes (e.g., many participants did not know why menstruation occurs) (Figure 1).

As part of its community trust-building strategy, TLP worked with women recruited to serve as local TLP ambassadors. These ambassadors encouraged participation among women in the community and helped plan the dates and locations of educational workshops, screening events, and results-delivery sessions. As a result, each screening event required extensive pre-collaboration, education, and planning with key stakeholders and women in the community.

### Mobile clinic set up and intake process

On the date of the pre-scheduled screening, TLP staff arrived in a truck at a rural community site to set up their mobile clinic exam room in a designated location, typically a school, church, or unoccupied public health clinic. The room required no electricity and could be set up in minutes. Exam equipment included a foldable exam table, battery-powered headlamps worn by examiners, disposable exam equipment (speculums and sampling kits), materials to ensure a sterile set-up, and cryotherapy equipment (a gas tank and cryogun) when treatment was needed.

**Figure 2:**
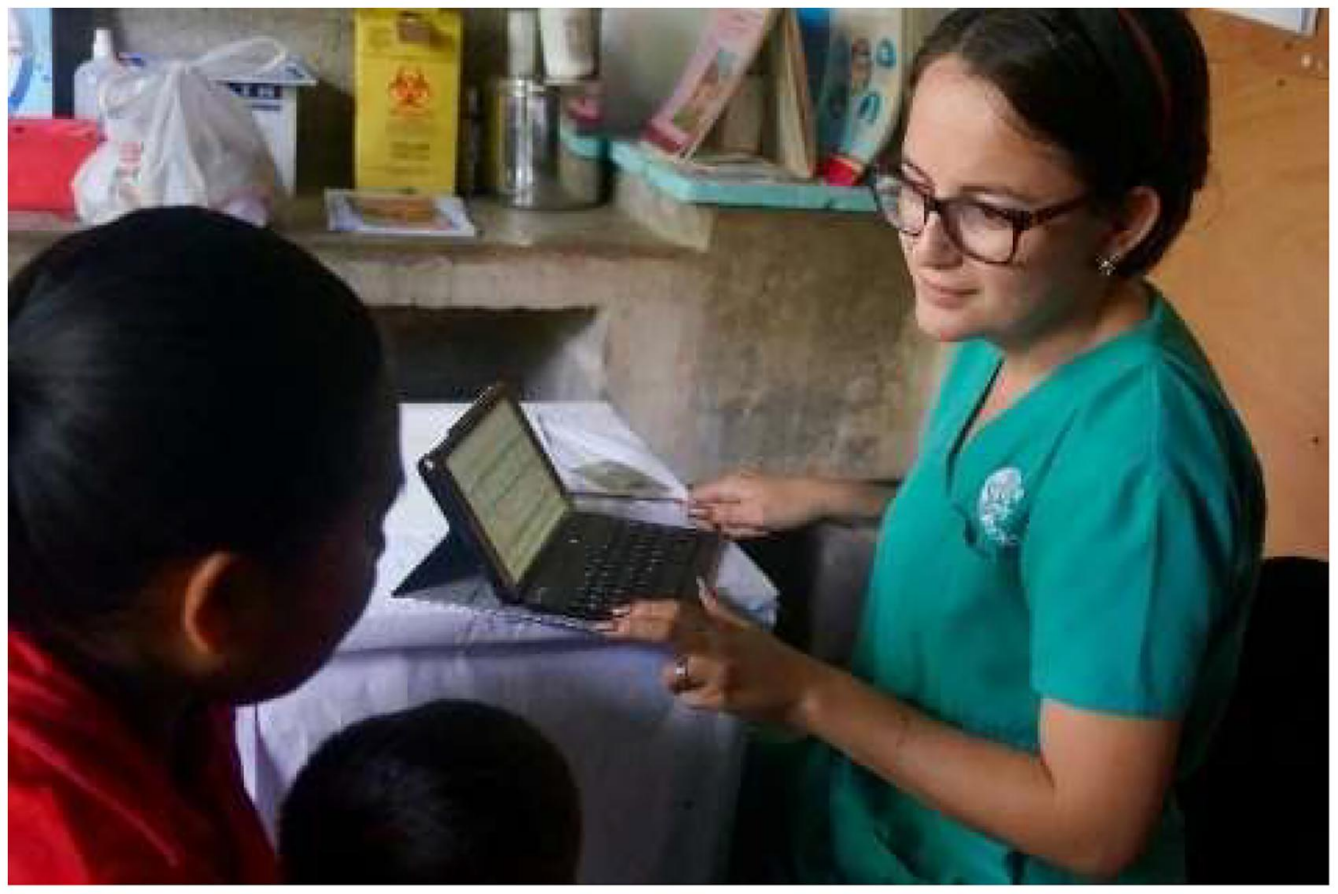
TLP private intake process. Photo credit: TLP 2019.

An intake interview was conducted with each client separately by a female TLP staff member. Observations of TLP screening events indicated that staff were attentive to the need for respect, privacy, and confidentiality throughout the intake and exam process. A female TLP staff member met with each woman individually in a private space to collect the necessary information prior to the exam. In addition to collecting health information required by government policies, the all-women staff clearly explained the procedure the client would receive, emphasized the privacy and confidentiality of the process, ensured that only new and sterile equipment was used, and explained that results would be provided either the same day (for VIA) or within two weeks (for Pap) (Figure 2).

During the intake process, TLP staff encouraged women to ask questions and spent time explaining and answering questions related to cervical cancer, HPV, the current screening procedure, and other reproductive health concerns. TLP team members not involved in the exam room continued the intake process and provided general education about cervical cancer to women waiting for exams, as well as to other women and girls in attendance (women often came with children or other female family members).

While initially using paper intake forms, TLP developed the capacity to collect data electronically over time using tablets. The tablets utilized a modified program to complete intake forms digitally. Each woman's intake information was saved on password-protected tablets, and at the end of each screening event, paper forms were submitted to the Ministry of Health. After returning to the office, TLP staff securely uploaded client information from the tablets into a password-protected Excel database and then cleared all personal health information from the tablets.

Screening exams were conducted in a private exam room with two female TLP staff members—one to conduct the exam and the other to assist with sterile set-up and to provide emotional support and information throughout the procedure. Each exam was performed with attention to the woman's needs, with staff explaining each step of the process. If cryotherapy was indicated, staff explained the procedure and the reason for treatment, giving clients the option to receive the procedure the same day or to return at a later date.

**Figure 3:**
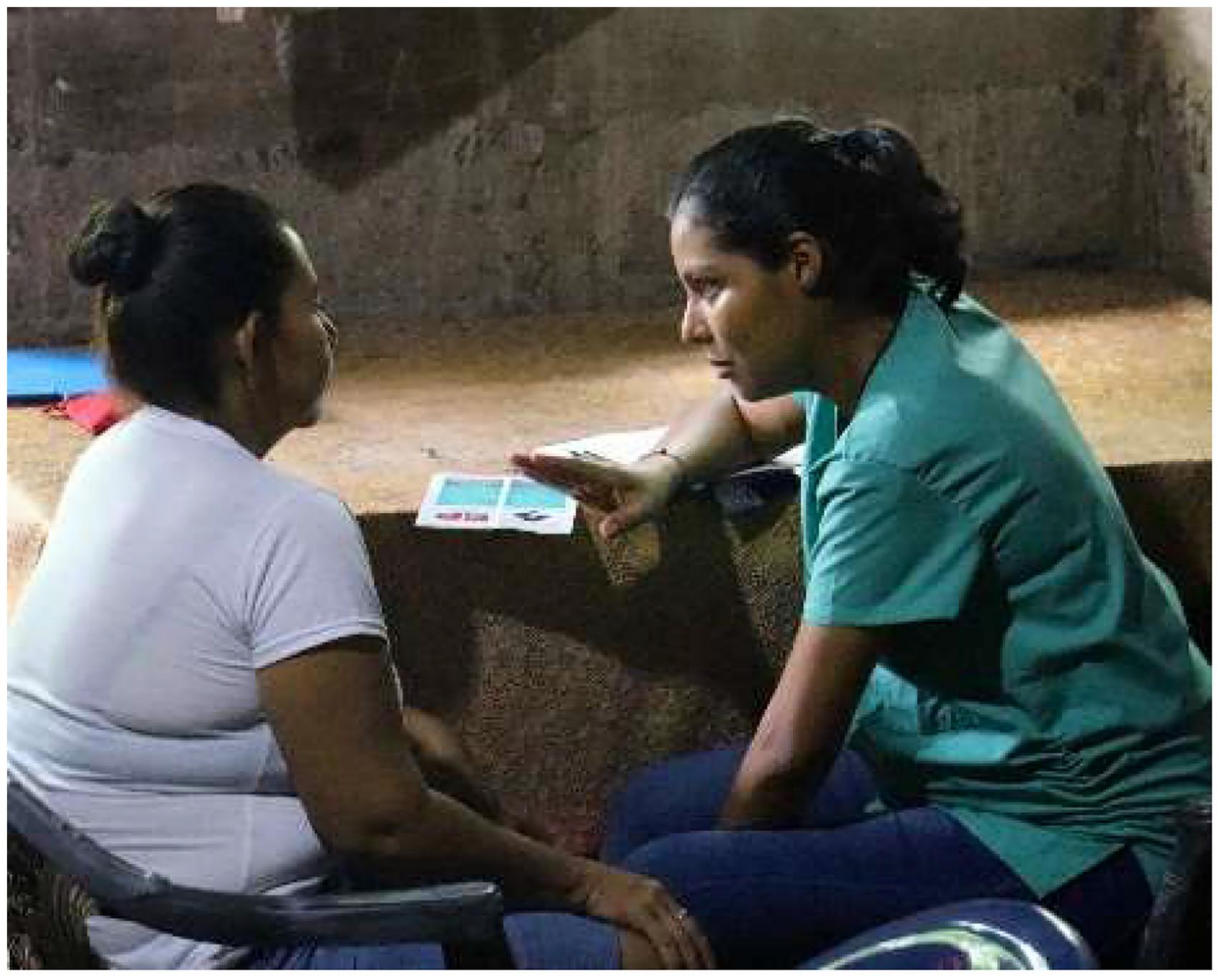
TLP director delivering individual results. Photo credit: TLP 2019.

Women who received Pap exams were asked how they preferred to receive their results: via the local health clinic, through a community leader, or by personal delivery from TLP staff on a pre-established date at a central community location. Most women chose personal delivery and attended results-delivery events to meet individually with TLP staff to receive and discuss their results. If women were not reached through their preferred method, TLP staff continued follow-up by contacting a community leader or TLP ambassador, or by reaching out directly to the woman via WhatsApp (Figure 3).

Importantly, results-delivery visits also included explanation of any vaginal infections or inflammation identified through Pap testing, medication, if needed, and explanations about how to take the medication or other steps to improve their gynocological health. In addition to providing results directly to women, TLP submitted all paper intake forms and Pap results to the local Ministry of Health. This ensured that women were registered within the Ministry of Health system and had a record of their exam available at their local health center.

### Continuance of care

The TLP model recognized the importance of following up with clients after screening to address questions or concerns and to deliver Pap results. In-person delivery of results by TLP's trained staff was the most frequently selected method of results delivery. This approach enabled staff to meet individually with each client to confidentially explain results and discuss next steps. While community ambassadors may have assisted with contacting clients, all results were delivered in sealed envelopes to ensure confidentiality. Importantly, TLP staff also used WhatsApp (a smartphone-based communication application widely used in Nicaragua) to deliver results when needed and to continue follow-up conversations with clients after screening.

The TLP database was central to tracking women's visits and follow-up needs, particularly for those placed on TLP's “watch list.” TLP leaders and staff regularly used the database to generate lists of women expected to attend follow-up screening or treatment events. If a woman did not attend, TLP staff contacted her directly or relied on a visit from a community ambassador to encourage follow-up. As a result, the TLP process involved multiple points of contact with clients, with the goal of increasing the likelihood that women would receive—and understand—the tests and procedures needed.

Importantly, TLP also established protocols for assisting clients who required further treatment or additional examinations. For women who qualified for cryotherapy but did not receive treatment on the day of the exam, or for those requiring colposcopy or biopsy, TLP arranged return visits to the community to provide follow-up care. While TLP had cryotherapy training and equipment from the outset of the program, in later years staff were also trained in colposcopy and acquired a mobile colposcope. For women requiring more advanced procedures, TLP facilitated referrals to public hospitals, assisted with appointment scheduling, and helped address transportation barriers.

### The Lily Project—outcomes

#### Women reached

Analysis of TLP data indicated that between 2015 and 2020, the organization conducted 19,949 exams through its screening events across the two regions. TLP clients came from more than 300 neighborhoods and communities across 19 of the 23 municipalities within the districts of Matagalpa and León. The median age of TLP clients was 34, and 96% were between ages 18 and 67. Approximately 90% of women reported living in rural areas. More than 60% of TLP clients had not completed primary education, further indicating the social vulnerability of this population and limited exposure to school-based health education.

#### Exam results

Of all women screened by TLP, 63% received a Pap test and 35% received VIA screening. A small proportion (2%) received both types of screening, potentially due to difficulties visualizing the cervix. Among VIA screenings, 92% were negative and 8% were positive for lesions. Among Pap tests, 87% were normal, 10% were abnormal, and 3% were indeterminate and required a repeat exam.

It should also be noted that regular training by gynecologists ensured that TLP staff were conducting technically high-quality exams. They also used a private lab, with established experience in Pap analyses. Only 3% of all of the Pap samples collected by TLP had to be repeated, some of which were due to inflammation and other issues. Thus, a relatively small number, if any, were due to sample collection issues.

Additionally, results indicated that approximately 15% of TLP clients who received a Pap exam had an infection (of those detectable by the exam), and 45% had moderate or severe inflammation. TLP clients had relatively low rates of the most common infection - bacterial vaginosis (11.4%).

#### Follow-up & treatment

For VIA screening, women received their results at the end of the exam and, when appropriate, were offered immediate cryotherapy or the option to return in two weeks for a separate cryotherapy appointment in a nearby location. TLP data indicated that approximately half of the women who were eligible for same-day cryotherapy received treatment that day. Interviews with TLP staff indicated that women who declined same-day treatment cited reasons such as not having time that day, needing to consult with a spouse, or not feeling prepared to receive treatment at that time.

For Pap results, TLP staff received the results from a private lab and contacted the women via WhatsApp (including the female ambassador in the community that coordinated with TLP) to let them know the time and place of the results delivery. Importantly, TLP included in the sealed envelope not only the Pap results but also any medication that was needed for detected infections and instructions on how to take it. This provided an additional incentive for women to receive their results and support for their broader gynecological health. If women could not attend the results meeting, they were able to elect to send someone on their behalf or have the envelop be left with the community collaborator to receive the envelop later. TLP had women sign upon receipt of the results, but this information was not aggregated into the database and is unable to be accessed. However, Pap results were delivered within the two-week goal to the vast majority of women directly and an unknown small percent indirectly through the community leader. The high delivery rate was facilitated by the rapid turn-around of the results before women could be lost to follow up and the provision of medications where needed.

The bigger challenge was the longer-term follow-up for those who did not accept cryotherapy treatment the same day or required an additional test or follow-up appointment. The latest data available indicated that among all women with abnormal results (Pap or VIA), 48% continued to be monitored by TLP (for a later exam or treatment), 22% received cryotherapy from TLP, and 12% were referred for treatment. Of those referred for follow-up treatment, five died due to advanced cervical cancer. The remaining 18% were lost to follow-up or not tracked in the data. *Women's Views of The Lily Project.*

While we were unable to conduct a formal evaluation of TLP or systematically assess women's perceptions of care quality, in 2019 TLP surveyed a small group of women (n = 79) who returned for a second screening 1–2 years later. Nearly half of these women (49%) reported returning to TLP because of how they were treated, using phrases such as “*me gusta”* (“I like it”) or “*son amables”* (“they are kind”). Another 32% reported returning because “*entregan resultados”* (“they deliver results”), and 19% because “*dan seguimiento”* (“they follow-up”). These findings illustrate multiple domains of cervical cancer screening quality that were meaningful to rural Nicaraguan women.

## Discussion

Cervical cancer remains a major public health challenge, particularly in the global South. While medical advances in prevention include the development of the HPV vaccine, most low-income countries do not have the capacity to support widespread vaccine implementation. Cervical cancer screening therefore remains essential for preventing and treating cervical lesions before they progress to advanced stages. However, delivering high-quality screening services to marginalized populations continues to be a major barrier to reducing cervical cancer mortality in LMICs.

In response to these challenges, and particularly as they apply to the rural Nicaraguan context, the Lily Project established a women-centered model of cervical cancer screening aimed at providing high-quality care for cervical exams in low-resource settings. Reflecting on the results presented above, we discuss both the potential successes of, and challenges faced by The Lily Project.

### Successful areas

#### Implementing women-centered mobile care

Conceptual and empirical reviews of women-centered care indicate that the term has most often been used to describe maternity-related services, particularly the provision of choice, control, and continuity during pregnancy and birth (20) (Brady et al., 2019). More recent work extends women-centered care to broader domains, emphasizing empowerment and relationships built on trust between providers and clients (21). Women-centered approaches also highlight the importance of accessible communication, non-technical language, and shared decision-making, positioning health care as a space for empowerment rather than solely for diagnosis and treatment (22–25).

The women-centered design of TLP was evident across nearly every aspect of its goals and implementation. In particular, the mobile clinic model was developed in direct response to needs expressed by women in rural communities and staff members' lived experience in these contexts. First, accessibility was prioritized by bringing care directly into rural communities rather than requiring women to travel to centralized facilities, thereby addressing barriers such as transportation, time, cost, and competing caregiving responsibilities.

Second, TLP's women-centered approach addressed quality-of-care needs articulated by women, defining quality as privacy, the use of disposable materials, timely delivery of results, and the provision of services by female staff. These conditions provided by TLP contrasted sharply with women's experiences in under-resourced public health clinics. Although formal satisfaction data were limited, qualitative observations and survey responses indicate that women valued respectful treatment, reliable results delivery, and follow-up care, all of which was completed by female staff members. The rapid turnaround time for results delivery and attention to other issues, like infections, that arose in Pap exams aimed to further meet women's quality-of-care needs in addition to facilitating future screenings/treatment.

Third, TLP explicitly embraced women's empowerment as central to cervical cancer prevention, consistent with feminist approaches to health care. Recruiting an all-women staff with lived experience in rural Nicaragua, coupled with ongoing training in empathy, listening, and consent, aimed to reduce hierarchical barriers between TLP providers and clients and foster reciprocal trust.

Finally, TLP use of private space (physical and digital with WhatsApp), where women could confidentially communicate and share their bodily experiences with TLP staff, was also provided women with safe spaces to make informed choices and engender control over their bodies. Another care element consistent with a feminist, women-centered care approach (22). This was important not just for cervical cancer screening but for other gynecological health issues, such as inflammation and infections, which were detected during the exams. TLP model ensured women could discuss these results with TLP staff and get referrals for treatment. This may be particularly important for the detection and treatment of bacterial vaginosis, which impacts an estimated one in four women across LMICs (26).

#### Incorporating education

**Figure 4:**
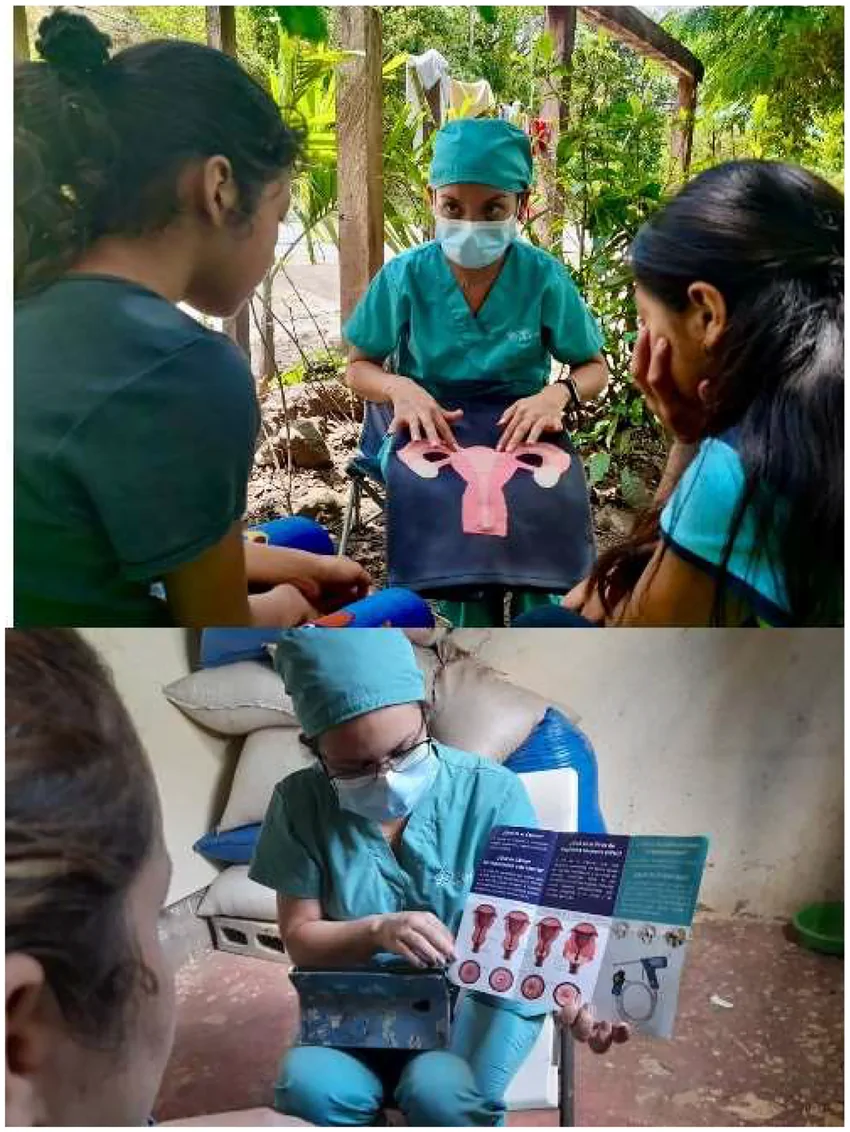
TLP educating girls and women. Photo credit: TLP 2019.

Education was a core component of TLP’s approach and was delivered at multiple stages, including community-based events, intake processes, examinations, results delivery and during the follow-up patient navigation process. The community events prior to the mobile clinics may have been particularly impactful, given past evidence that suggests community group “charlas” and workshops by health centers promoted greater cervical cancer knowledge, and by extension, understanding of the purpose of the screenings (27). In addition to community education events, TLP staff also spent significant time addressing individual women’s concerns and providing continued education on sexual health and related issues during and after the screening events. For example, the top photo below illustrates pre-event education workshops, while the bottom photo is of the education happening during the intake process before each exam (Figure 4).

#### Community collaboration

Research and on-the-ground experiences in cervical cancer prevention highlight the importance of addressing community-level factors and collaborations among multiple partners when designing interventions in LMIC settings (28). TLP's success in engaging government and community support highlights the importance of multi-level collaboration in fragmented and underfunded health systems. Though TLP was a non-governmental organization, it effectively engaged national, regional, and local government institutions by positioning itself as a complementary partner rather than a competitor of the Ministry of Health. This facilitated both access to communities and connections with higher-level services needed for referrals. Community-level collaborators/ambassadors were also essential in building trust and disseminating information about cervical cancer screenings and in recontacting women who may have been otherwise lost to follow-up.

#### Challenges and limitation

TLP model also faced several limitations. First, it should be noted that they were working within the Ministry of Health guidelines for cervical cancer prevention, including using their intake forms and providing the screening tests dictated by the state. While this enabled TLP to work in communities where the Ministry of Health was present, it reduced their ability to use newer methods such as HPV testing.

Second, reliance on non-physician providers limited the range of procedures that could be offered during screening events, although this was mitigated through referral systems and partnerships with physician specialists who would attend the mobile clinics several times a year to conduct colposcopies, biopsies as advanced mobile procedures.

While TLP was very successful in short-term follow-up for delivery of results, longer-term patient navigation was a challenge. They had an established “watch list” and follow-up system using WhatsApp and connecting with community ambassadors, but still had an approximate 18% loss to follow-up rate. This is an estimate based on the existing data and may have occurred for several reasons, including: regularly changing cell phone numbers of their clients, errors in the data, or women refusing to return to follow-up visits and/or provide contact information. However, this loss to follow up rate is quite low compared to a pilot HPV-testing program in Nicaragua that had high self-testing uptake rates (97%) but lost almost half of the HPV-positive women (46%) before the required follow-up cervical exam. Of those who completed the follow up exam and were positive, another 47% were lost to follow-up before treatment (9).

Finally, increasing restrictions on NGO funding and operations by the Nicaraguan President and Vice President in their ongoing consolidation of power constrained TLP's ability to continue service delivery and ultimately forced them to close. This illustrates the precarity of NGOs in the broader context of cervical cancer prevention, like in other public health areas.

## Conclusion

Despite these limitations and challenges, The Lily Project provides a model of a women-centered approach to cervical cancer prevention that could be replicated in other low-income, rural settings. While the technical aspects of the screenings, such as Pap and VIA may be less ideal than HPV testing, the goal of TLP was to provide the necessary cervical screenings (which are needed even if HPV testing is done) in a way that is women-centered and provides attention to quality of care issues such as: privacy, respect, female providers, education and informed, reliable delivery of results. The stakeholder collaboration approach is worth noting as a way of bringing multiple sources of health care and support to women for the prevention of cervical cancer.

The Lily Project provides evidence that it is possible to provide women-centered, high-quality care and culturally acceptable cervical exams in challenging conditions and among hard-to-reach populations. The future of cervical cancer screening should combine lessons learned from this and other innovative approaches to prioritize women-centered approaches to screening care and follow-up, in addition to promoting HPV vaccine uptake, to reduce cervical cancer in low-income settings. Further, given evidence from Latin America (including Nicaragua) that women who have had a recent doctor's visit are more likely to have had a recent Pap smear (29), addressing women's access to quality health care more broadly is an important next step for cervical cancer prevention in Nicaragua and Latin America as a whole.

## Data Availability

The original contributions presented in the study are included in the article/Supplementary Material, further inquiries can be directed to the corresponding author.

## References

[1] WHO. Cervical Cancer [Internet]. Geneva: World Health Organization (2024). Available online at: https://www.who.int/news-room/fact-sheets/detail/cervical-cancer (Accessed August 19, 2025).

[2] Han J Zhang L Chen Y Zhang Y Wang L Cai R. Global HPV vaccination programs and coverage rates: a systematic review. eClinicalMed. (2025) 84:103290. 10.1016/j.eclinm.2025.103290 PMC12179740 40547442

[3] Lei J Ploner A Elfström KM Wang J Roth A Fang F. HPV Vaccination and the risk of invasive cervical cancer. N Engl J Med. (2020) 383(14):1340–8. 10.1056/NEJMoa1917338 32997908

[4] De Vuyst H Claeys P Njiru S Muchiri L Steyaert S De Sutter P. Comparison of pap smear, visual inspection with acetic acid, human papillomavirus DNA-PCR testing and cervicography. Int J Gynaecol Obstet. (2005) 89(2):120–6. 10.1016/j.ijgo.2005.01.035 15847874

[5] Srinath A Van Merode F Rao SV Pavlova M. Barriers to cervical cancer and breast cancer screening uptake in low- and middle-income countries: a systematic review. Health Policy Plan. (2022) 38(4):509–27. 10.1093/heapol/czac104 PMC10089064 36525529

[6] WHO. Global Strategy to Accelerate the Elimination of Cervical Cancer as a Public Health Problem. 1st ed Geneva: World Health Organization (2020). p. 1 p.

[7] Bray F Ferlay J Soerjomataram I Siegel RL Torre LA Jemal A. Global cancer statistics 2018: gLOBOCAN estimates of incidence and mortality worldwide for 36 cancers in 185 countries. CA Cancer J Clin. (2018) 68(6):394–424. 10.3322/caac.21492 30207593

[8] Pilleron S Cabasag CJ Ferlay J Bray F Luciani S Almonte M. Cervical cancer burden in Latin America and the Caribbean: where are we? Int J Cancer. (2020) 147(6):1638–48. 10.1002/ijc.32956 32150288

[9] Holme F Maldonado F Martinez-Granera OB Rodriguez JM Almendarez J Slavkovsky R. HPV-based cervical cancer screening in Nicaragua: from testing to treatment. BMC Public Health. (2020) 20:495. 10.1186/s12889-020-08601-z 32295562 PMC7161152

[10] Mosquera I Barajas CB Zhang L Lucas E Benitez Majano S Maza M. Assessment of organization of cervical and breast cancer screening programmes in the Latin American and the Caribbean states: the CanScreen5 framework. Cancer Med. (2023) 12(19):19935–48. 10.1002/cam4.6492 37768035 PMC10587918

[11] Pan American Health Organization. Cervical Cancer Prevention and Control Programs: A Rapid Assessment in 12 Countries of Latin America. Washington, D.C: PanAmerican Health Organization (2010). Available from: Available online at: https://www.paho.org/en/documents/paho-cervical-cancer-prevention-and-control-programs-rapid-assessment-12-countries-latin (Accessed May 22, 2026).

[12] Campos NG Mvundura M Jeronimo J Holme F Vodicka E Kim JJ. Cost-effectiveness of HPV-based cervical cancer screening in the public health system in Nicaragua. BMJ Open. (2017) 7(6):e015048. 10.1136/bmjopen-2016-015048 28619772 PMC5623348

[13] Allanson E Schmeler K. Cervical cancer prevention in low and middle income countries. Clin Obstet Gynecol. (2021) 64(3):501–18. 10.1097/GRF.0000000000000629 34120126 PMC8324570

[14] Daniel D Wickerham A Fitzgerald EA Kue J. Interventions to eliminate cervical cancer in Nicaragua: an integrative review of the literature. Hisp Health Care Int. (2023) 21(2):104–13. 10.1177/15404153221081280 35369782

[15] Lion KC Prata N Stewart C Adolescent childbearing in Nicaragua: a quantitative assessment of associated factors. Int Perspect Sex Reproduct Health. 2009;35(2):91–6. 10.1363/ifpp.35.091.09 19620093

[16] Parker JJ Veldhuis CB Hughes TL Haider S. Barriers to contraceptive use among adolescents in two semi-rural Nicaraguan communities. Int J Adolesc Med Health. (2020) 32(5):2017–28. 10.1515/ijamh-2017-0228 30939115

[17] Steppe JD Blake BJ Dyal MA Bailey TS Porter KJ Thompson J Conducting a health education needs assessment in rural Nicaragua. Health Educ J. (2020) 79(5):UNSP 0017896919896615. 10.1177/0017896919896615

[18] Gillberg C Jones G. Feminism and healthcare: toward a feminist pragmatist model of healthcare provision. In: Liamputtong P, editor. Handbook of Research Methods in Health Social Sciences. Singapore: Springer (2019). p. 205–22. 10.1007/978-981-10-5251-4_64

[19] Horiuchi S Kataoka Y Eto H Oguro M Mori T. The applicability of women-centered care: two case studies of capacity-building for maternal health through international collaboration. Jpn J Nurs Sci. (2006) 3(2):143–50. 10.1111/j.1742-7924.2006.00060.x

[20] Brady S Lee N Gibbons K Bogossian F. Woman-centred care: an integrative review of the empirical literature. Int J Nurs Stud. (2019) 94:107–19. 10.1016/j.ijnurstu.2019.01.001 30951986

[21] Brady S Gibbons KS Bogossian F. Defining woman-centred care: a concept analysis. Midwifery. (2024) 131:103954. 10.1016/j.midw.2024.103954 38364459

[22] Andrist L. A feminist model for women’s health care. Nurs Inq. (1997) 4(4):268–74. 10.1111/j.1440-1800.1997.tb00113.x 9437964

[23] Sen G. Gender equality and women’s empowerment: feminist mobilization for the SDGs. Glob Policy. (2019) 10(S1):28–38. 10.1111/1758-5899.12593

[24] Sen G Iyer A Chattopadhyay S Khosla R. When accountability meets power: realizing sexual and reproductive health and rights. Int J Equity Health. (2020) 19(1):111. 10.1186/s12939-020-01221-4 32635915 PMC7341588

[25] Worrell J. Feminist interventions: accountability beyond symptom reduction. Psychol Women Q. (2001) 25(4):335–43. 10.1111/1471-6402.00033

[26] Peebles K Velloza J Balkus JE McClelland RS Barnabas RV. High global burden and costs of bacterial vaginosis: a systematic review and meta-analysis. Sex Transm Dis. (2019) 46(5):304–11. 10.1097/OLQ.0000000000000972 30624309

[27] Rees HD Lombardo AR Tangoren CG Meyers SJ Muppala VR Niccolai LM. Knowledge and beliefs regarding cervical cancer screening and HPV vaccination among urban and rural women in león, Nicaragua. PeerJ. (2017) 5:e3871. 10.7717/peerj.3871 29085745 PMC5660604

[28] Agurto I Arrossi S White S Coffey P Dzuba I Bingham A. Involving the community in cervical cancer prevention programs. Int J Gynaecol Obstet. (2005) 89(S2):S38–45. 10.1016/j.ijgo.2005.01.015 15823265

[29] Soneji S Fukui N. Socioeconomic determinants of cervical cancer screening in Latin America. Revista Panamericana de Salud Pública. (2013) 33:174–82. 10.1590/S1020-49892013000300003 PMC3724344 23698136

